# Retraction: Casticin potentiates TRAIL-induced apoptosis of gastric cancer cells through endoplasmic reticulum stress

**DOI:** 10.1371/journal.pone.0322907

**Published:** 2025-04-15

**Authors:** The PLOS One Editors

After this article [1] was published, concerns were raised regarding results presented in Figs 2 and 4-7.

Specifically:

- In the Fig 2H PARP panel, when contrast levels are adjusted, there appear to be regions surrounding lanes 1, 2, 4, and 5 where the background does not match the overall background of the panel.
- The following results appear similar despite representing different experimental results:
  ◦ Fig 4A DR4 panel lane 3 and lane 4.
  ◦ Fig 5B DR5 panel lane 3 and lane 5.
  ◦ Fig 6A DR4 panel lanes 2-3 and lanes 4-5.
- The following panels appear similar despite representing different experimental results:
  ◦ Fig 6C CHOP panel in [1] and Fig 4D GADD153 panel in [2].
  ◦ Fig 6C DR4 panel in [1] and Fig 4B β-actin panel in [2].
  ◦ Fig 7C DR5 panel in [1] and Fig 7B DR5 panel in [3], retracted in [4].
  ◦ Fig 7C CHOP panel in [1] and Fig 7B CHOP panel in [3,4].
  ◦ Fig 7C β–actin panel in [1] and Fig 7B β–actin panel in [3,4].
- There appear to be one or more vertical discontinuities in the following panels in [1]:
  ◦ Fig 5B β-actin panel.
  ◦ Fig 6A GRP78 panel.
  ◦ Fig 6A DR4 panel.

The authors did not respond to editorial requests for a response and underlying data.

After publication of [1], the BGC-823, SGC-7901, and MGC-803 cell lines reported in [1] were identified as contaminated cell lines and are potential derivatives of HeLa [5–8].

In light of the above unresolved concerns, which undermine the reliability of the reported results and conclusions, the *PLOS One* Editors retract this article.

All authors either did not respond directly or could not be reached.
